# Contribution of a Novel Pertussis Toxin-Like Factor in Mediating Persistent Otitis Media

**DOI:** 10.3389/fcimb.2022.795230

**Published:** 2022-03-11

**Authors:** Longhuan Ma, Colleen Sedney, Yang Su, Kalyan K. Dewan, Bodo Linz, Eric T. Harvill

**Affiliations:** ^1^ Department of Infectious Diseases, College of Veterinary Medicine, University of Georgia, Athens, GA, United States; ^2^ Department of Biochemistry and Molecular Biology, University of Georgia, Athens, GA, United States

**Keywords:** chronic otitis media (COM), *Bordetella pseudohinzii*, PTx-like toxin, T cell function, middle ear

## Abstract

Chronic otitis media (COM) is the long-term infection and inflammation of the middle ears typically caused by upper respiratory tract pathogens that are able to ascend the Eustachian tube. Our understanding of contributing factors is limited because human otopathogens cannot naturally colonize or persist in the middle ears of mice. We recently described a natural COM in mice caused by *Bordetella pseudohinzii* and proposed this as an experimental system to study bacterial mechanisms of immune evasion that allow persistent infection of the middle ear. Here we describe a novel pertussis toxin (PTx)-like factor unique to *B. pseudohinzii*, apparently acquired horizontally, that is associated with its particularly efficient persistence and pathogenesis. The catalytic subunit of this toxin, PsxA, has conserved catalytic sites and substantial predicted structural homology to pertussis toxin catalytic subunit PtxA. Deletion of the gene predicted to encode the catalytic subunit, *psxA*, resulted in a significant decrease in persistence in the middle ears. The defect was not observed in mice lacking T cells, indicating that PsxA is necessary for persistence only when T cells are present. These results demonstrate the role of a novel putative toxin in the persistence of *B. pseudohinzii* and its generation of COM. This PsxA-mediated immune evasion strategy may similarly be utilized by human otopathogens, *via* other PTx-like toxins or alternative mechanisms to disrupt critical T cell functions necessary to clear bacteria from the middle ear. This work demonstrates that this experimental system can allow for the detailed study of general strategies and specific mechanisms that otopathogens use to evade host immune responses to persist in the middle ear to cause COM.

## Introduction

Otitis media (OM) is the infection and inflammation of the middle ears, and a major reason for doctor visits and antibiotic prescriptions among children (Schilder et al., 2016). Many causative agents of OM begin as colonizers of the nasopharynx and then ascend the Eustachian tube *via* poorly understood mechanisms. While most infections are resolved by host immune mechanisms, some result in persistent infections of the middle ear. A variety of human pathogens have been identified as responsible for such infections, including *Streptococcus pneumoniae, Haemophilus influenza*, and *Moraxella catarrhalis*, among others (Palmu et al., 2004; Massa et al., 2009). Many of these infections result in pain, inflammation, and fever, sometimes leading to ear drum perforation and hearing loss (Schilder et al., 2016). However, these human pathogens do not naturally establish infections in murine models, a limitation overcome by injecting large numbers of human pathogens directly into the middle ears of mice to study aspects of the severe inflammation that results. But this approach confounds the ability to study bacterial factors and mechanisms that mediate aspects of efficient colonization and long-term persistence in the middle ear.

We have previously described the use of *Bordetella pseudohinzii*, a bacterial species that efficiently colonizes and persists in the middle ears of mice as an experimental model for natural OM progression (Dewan et al., 2019). *B. pseudohinzii* is a recently described species (Ivanov et al., 2016) that, when introduced in very small numbers to the external nares, naturally and efficiently ascends the Eustachian tube, colonizes, and persists in the middle ears of mice for over three months Dewan et al., 2019), apparently for the life of the animal. In addition to its strengths as a model for OM, *B. pseudohinzii* has also been investigated as an upper respiratory tract pathogen that forms biofilms (Clark et al., 2016) and is cytotoxic to ciliated epithelial cells (Perniss et al., 2018). In contrast to *B. pseudohinzii*, a related species, *B. bronchiseptica*, can highly efficiently colonize both nasal cavity and middle ears of mice, and persists for life in the noses but is cleared from the middle ears by processes that involve adaptive immunity (Dewan et al., 2019; Dewan et al., 2022).

Here we investigate the possible mechanisms underlying the immune evasion that allows *B. pseudohinzii* to persist in the middle ear. Genome comparisons revealed numerous putative virulence factor genes unique to *B. pseudohinzii*, of which a set of pertussis toxin (PTx)-like genes were selected for further analysis. Pertussis toxin (PTx) is specifically produced by *B. pertussis* and is a major contributor to the disease process and immunomodulatory effects of *B. pertussis.* PTx is an AB_5_ toxin that is made of two major components, the A-promoter (S1 or PtxA subunit) and the B-oligomer (S2-S5 or PtxB-PtxE subunits). PTx-like toxins are characterized by their ADP-ribosyltransferase activities and include toxins produced by several species, such as *E. coli* and *Salmonella spp*, among others (Littler et al., 2017; Tamamura et al., 2017). PTx hydrolyzes NAD to ADP-ribose and nicotinamide as well as transfer the ADP-ribose to the target proteins (Krueger and Barbieri, 1995). Here we report a set of PTx-like toxin genes in the genome of *B. pseudohinzii* that encode a putative novel toxin which is annotated as Pseudohinzii toxin (PSx). To examine the role of PSx in persistence, an in-frame deletion mutant of the gene encoding the putative catalytic component, psxA, was generated. This deletion of *psxA* resulted in a mutant strain that failed to efficiently persist in the middle ears of mice. The predicted structure of this PTx-like toxin subunit and its role in modulating the host immune response in the middle ears of mice suggest PSx is a novel immunomodulatory agent disrupting immune mediated clearance of bacteria from the middle ear.

## Materials And Methods

### Bacterial Strains and Growth

*B. pseudohinzii* 8-296-03 (Ivanov et al., 2016), and *B. bronchiseptica* RB50 (Cotter et al., 1998) have been previously described. Both bacteria were grown and maintained on Bordet-Gengou (BG) agar (Becton Dickson) supplemented with 10% defibrinated sheep’s blood (HemoStat). For mouse inoculations, the bacterial strains were grown at 37°C, with shaking at 200 rpm, to mid-log phase in Stainer Scholte (SS) liquid broth (Stainer and Scholte, 1970). Numbers of bacterial colony forming units (CFU) were estimated by measuring the optical density at 600 nm, validated by dilution in phosphate buffered saline (PBS), plating on BG agar and counting viable colonies after incubation for 2 days at 37°C.

### Mutant Generation

The allelic exchange vector pSS4245 was used for the generation of deletion mutants. Briefly, ∼1 kb of DNA flanking each end of gene *psxA* was PCR amplified with primers from IDT (Upstream forward: 5’-AGGGCGGCCGCACTAGGGGTTGAGTTCGCGGGCGAAACCAG-3’; Upstream reverse: 5’-TGGCTGCCAGTTATTGACGCATACCCACGCCATTCCTGCTATG-3’; Downstream forward: 5’-TGGCGTGGGTATGCGTCAATAACTGGCAGCCACGATATGGTG-3’; Downstream reverse: 5’-GATCTGTACACCTAGGGGACGATGAGTACACGCGAATAC-3’), joined and inserted into the allelic exchange vector, pSS4245, by PIPE cloning (Plasmid vector amplification primers: Forward primer: 5’-CTAGTGCGGCCGCCCTAGCATAGG-3’; Reverse primer: 5’-CCTAGGTGTACAGATCCGGACCTGC-3’) (Klock and Lesley, 2009). The construct was verified by sequencing, transformed into *E. coli* SM10λ*pir*, and transferred into the parental *B. pseudohinzii* 8-296-03 by mating. Colonies containing the integrated plasmid were selected and incubated on BG agar to stimulate allelic exchange by homologous recombination. Emerging colonies were screened by PCR for replacement of the wildtype by the mutant allele and confirmed by Sanger sequencing.

### Genomic and Protein Structure Analysis

Total protein sequences were extracted from the NCBI archive for *B. bronchiseptica* RB50 (RefSeq assembly accession: GCF_000195675.1), *B. pertussis* Tohama I (GCF_000195715.1)) and *B. pseudohinzii* HI4681 (GCF_001698185.1) and 8-296-03 (GCF_000657795.2). Similarities between *B. bronchiseptica* and *B. pseudohinzii* proteins were estimated as H values using mGenomeSubtractor (Shao et al., 2010). H values were determined as the highest BLASTp identity score (i), multiplied by the matching sequence length (lm) divided by the query length (lq) as H = i x (lm/lq). Based on our previous work (Rivera et al., 2019), genes encoding proteins with an H value < 0.5 were considered absent from the genome. The genomes of *B. pseudohinzii* HI4681 (Spilker et al., 2016) and 8-296-03 (Ivanov et al., 2016) were subjected to pairwise tBLASTx searches against the genomes of *B. pertussis* Tohama I and of *B. bronchiseptica* RB50 (Parkhill et al., 2003), respectively. Pairwise comparisons were visualized using the Artemis Comparison Tool (Carver et al., 2005). The comparisons revealed conserved gene synteny of the entire pertussis toxin operon (*ptx* and *ptl* genes), with the exception of *psxB* that was duplicated in *B. pertussis* and *B. bronchiseptica* genomes. The comparisons also showed that the *B. pseudohinzii psx* operon was inserted at different genomic location compared to the classical *Bordetella* species.

Pertussis toxin protein identity values were determined from pairwise BLASTp comparisons, nucleotide identities of the individual genes were determined using BLASTn. Pairwise nucleotide identity (ANI) of the complete genomes was calculated at https://www.ezbiocloud.net/tools/ani (Yoon et al., 2017). The protein sequences of *B. bronchiseptica, B. pertussis*, and *B. pseudohinzii* toxins were retrieved from NCBI. Protein sequence alignment was conducted by Geneious R10 v 2021.1.1 using global alignment with free end gaps and cost matrix as BLOSUM62. *De novo* and comparative structure predictions were conducted using the Robetta online server (https://robetta.bakerlab.org/). The crystal structure of *B. pertussis* PtxA was retrieved from PDB. The predicted structure of *B. pseudohinzii* PsxA was aligned with *B. pertussis* PtxA and the RMSD value calculated by Pymol v2.5.2.

### Mouse Experiments

Four- to six-week-old mixed sex C57BL/6J (000664), B6.129S7-Rag1^(tm1Mom)/J (002216), B6.129P2-Tcrb^(tm1Mom)Tcrd^(tm1Mom)/J (002122) mice were procured from The Jackson Laboratory (Bar Harbour, ME) and bred in the Harvill laboratory mouse colony (University of Georgia, GA). All mice were maintained in specific pathogen-free facilities, and all experiments were conducted following institutional guidelines. Mice were lightly sedated with 5% isoflurane (Pivetal) and inoculated (150 CFU in 5 μL PBS) by pipetting the inoculum as droplets onto their external nares to be inhaled. At the indicated timepoints mice were euthanized *via* CO_2_ inhalation. Blood was drawn by cardiac puncture, and the organs were excised. To quantify bacterial numbers colonizing the middle ears, tissues were homogenized in 1 ml PBS, serially diluted, and plated on BG agar. Colonies were counted following incubation for two days at 37°C.

### Biofilm Assays

*B. pseudohinzii* strain 8-296-03 (Ivanov et al., 2016) and the confirmed *psxA* mutant were grown for 48 hours at 37°C on Bordet-Gengou (BG) agar (Becton Dickson) supplemented with 10% defibrinated sheep’s blood (HemoStat). The bacterial strains were then grown at 37°C, with shaking at 200 rpm, to mid-log phase in Stainer Scholte (SS) liquid broth (Stainer and Scholte, 1970). Cultures were diluted with sterile 1x PBS to OD_600 _= 0.1. These cultures were serially diluted to a final dilution of 10^-6^ of the original OD_600 _= 0.1 culture. 10 μl of each strain was added to a well of a 96-well plate containing 90 μl of -Mg^2+^ -Ca^2+^ PBS. Three technical repeats were performed. Plates were incubated for 48 hours at 37°C, after which the wells were washed three times with DI water. Following the washes, 125 μl of 0.05% crystal violet was added to each well and the plate was incubated for 15 minutes at room temperature. Excess crystal violet was removed and 200 μl of 200 proof EtOH was added to each well. Absorbance of each well was read immediately at 540 nm (BMG Labtech CLARIOstar multi-mode microplate reader).

### Statistics

For experiments determining differences in bacterial loads in the organs of mice, the following statistical analysis were performed using GraphPad PRISM (GraphPad Software, Inc): Two-tailed unpaired Student t-tests, One-way ANOVA and Two-way ANOVA were used to determine statistical differences between two groups. The specific test used is indicated in the Figure Legends.

### Ethics Statement

This study was carried out in accordance with the recommendations in the Guide for the Care and Use of Laboratory Animals of the National Institutes of Health. The protocol was approved by the Institutional Animal Care and Use Committees at The University of Georgia at Athens, GA (A2016 02-010-A13 Host-Pathogen Interactions, A2016 07-006-A5 Breeding Protocol). Mice were consistently monitored for signs of distress over the course of the experiments to be removed from the experiment and euthanized using carbon dioxide inhalation to prevent unnecessary suffering.

## Results

### Timecourse of Colonization of Middle Ears

**Figure 1:**
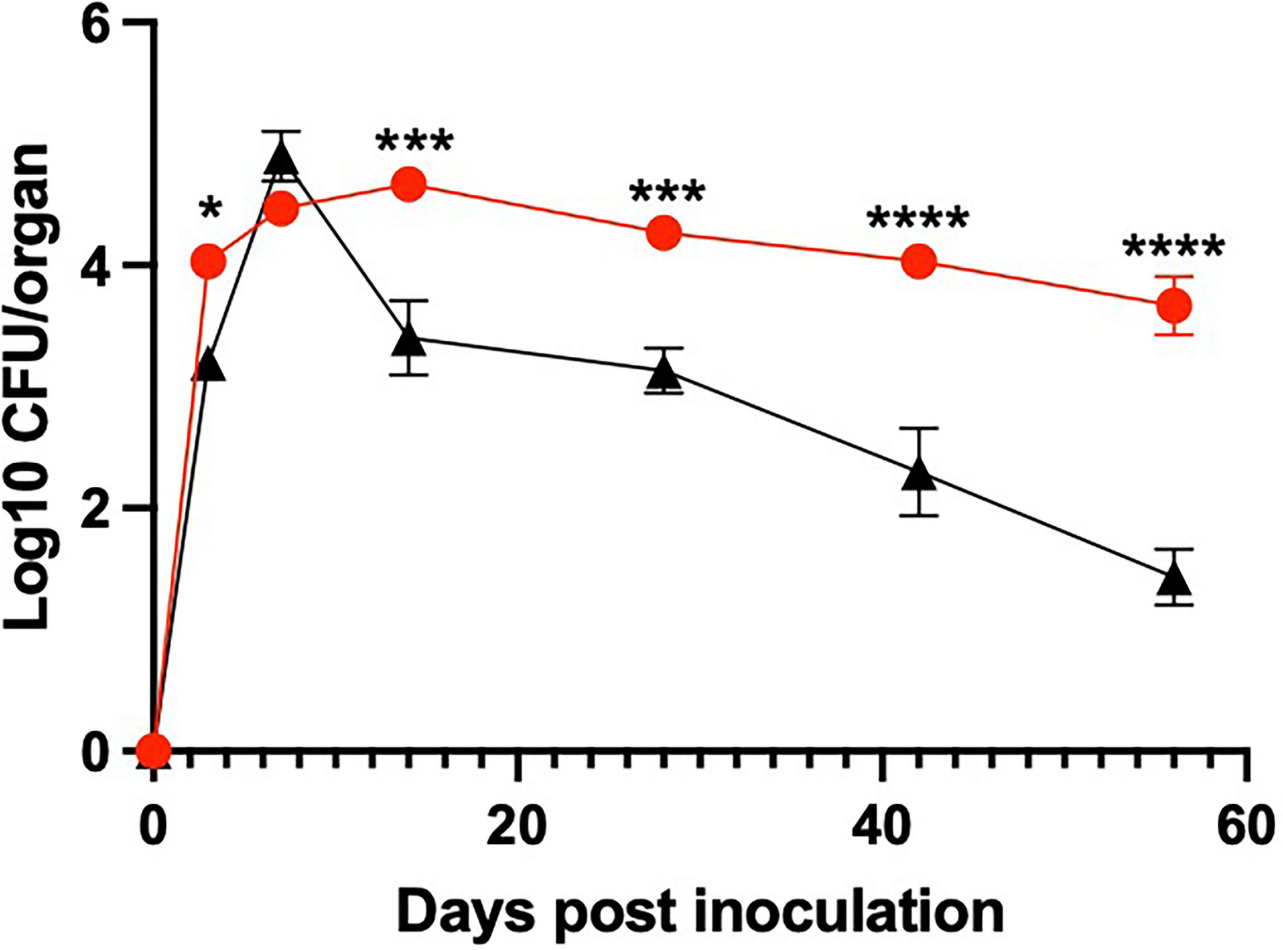
*B. pseudohinzii* efficiently colonizes and persists in the middle ears. Log10 of CFU recovered on days 3, 7, 14, 28, 42 and 56 post inoculation from the middle ears of mice inoculated with either *B. bronchiseptica* (black) or *B. pseudohinzii* (red) bacteria (n=4 per strain per timepoint). Error bar shows the standard error of mean. Statistical significance was calculated by using Two-way ANOVA. *p <  0.0332, ***p < 0.0002, ****p < 0.0001.

To evaluate the ability of *B. bronchiseptica* and *B. pseudohinzii* to infect and persist in the middle ears of mice, groups of C57BL/6 mice were inoculated on the external nares with 5 μl of PBS containing a calculated 150 CFU of either wild-type (WT) *B. bronchiseptica* or WT *B. pseudohinzii.* Bacterial load in the middle ears was evaluated at 3, 7, 14, 28, 42, and 56 days post inoculation (dpi) (**Figure 1**). Within 3 dpi, both middle ears of all mice had become colonized with thousands of the respective bacteria, and numbers continued to rise to over 10,000 CFU by 7 dpi, over fifty-times the inoculation dose delivered to the distal nares. The great consistency of rapid colonization and growth indicate that both species are highly efficient in moving from the nasal cavity, up the Eustachian tube to colonize and grow in the middle ears of mice. Mice infected with *B. bronchiseptica* experienced a steep decline (>95% reduction) in bacterial numbers between 7 and 14 dpi. This decline continued to 56 dpi, and *B. bronchiseptica* was cleared from the middle ears by 100 dpi (Dewan et al., 2022) by the generation of a robust adaptive immune response (Dewan et al., 2022). In contrast, *B. pseudohinzii* persisted in the middle ears at high numbers, approximately 10,000 CFU, until at least day 100, the end of this timecourse, with no apparent decline, suggesting infection is chronic, potentially life-long (**Figure 1** and data not shown). This contrast in the ability to persist in the face of adaptive immunity suggests *B. pseudohinzii* has specialized mechanisms to avoid immune-mediated clearance that *B. bronchiseptica* lacks.

### Differential Genome Content

**Figure 2:**
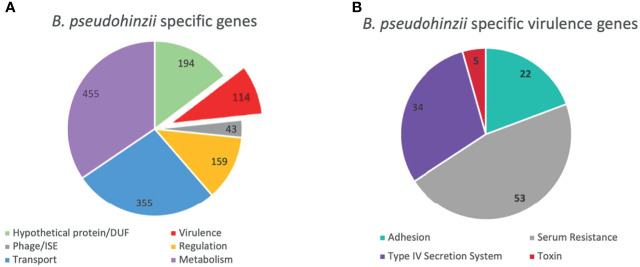
Unique genes present in *B. pseudohinzii* but not in *B. bronchiseptica*. **(A)** 1319 genes expressed in the genome of *B. pseudohinzii* that are lacking in the genome of *B. bronchiseptica* organized by functional category. **(B)** 114 virulence factors genes unique to the genome of *B. pseudohinzii* (from **A**) organized by function.

To assess possible mechanisms that mediate evasion of host immune clearance in the middle ears, the genomes of *B. pseudohinzii* and *B. bronchiseptica* were compared to identify genes particular to the former. The genome of *B. pseudohinzii* contains 4,168 coding genes and 30 pseudogenes, while *B. bronchiseptica* contains 4,768 coding genes and 13 pseudogenes. Utilizing mGenomeSubtractor on annotated genomes provided by NCBI, 1,319 genes were identified that are uniquely present in the genome of *B. pseudohinzii* that are lacking in *B. bronchiseptica* (**Figure 2A**). The annotated genes fall within the categories of metabolism (455), transport (355), regulation systems (159), virulence (114), domains of unknown function (194), and insertion sequences (43). Reasoning that a mechanism that allows for immune evasion and persistence of *B. pseudohinzii* might have homology to a known virulence factor, the 114 genes annotated as related to “virulence” and uniquely present in *B. pseudohinzii* (**Figure 2B**) were selected for further analysis. Of these, one locus of genes stood out as particularly promising based on its weak, but noticeable homology to a known immunomodulatory factor of *B. pertussis*, Pertussis toxin (PTx).

PTx is encoded in a set of genes apparently acquired by an ancestor of the three classical *Bordetella* species (*B. pertussis, B. bronchiseptica*, and *B. parapertussis*), and not shared by any other *Bordetella* species. The PTx-encoding locus of the classical *Bordetella* species and the PSx-encoding locus unique to only *B. pseudohinzii* share low homology, different gene numbers and different locations, contributing to confidence they were not acquired vertically from the last common ancestor. Their operons are composed of five and four genes each, respectively, with both *ptxB* and *ptxC* of the PTx locus sharing homology with *psxB/C* of the PSx locus. Further, the homology between the individual components of the PTx and PSx loci are weak relative to that of their shared core genome (80.34%) with H values between H=0.224 (PsxD) and H=0.500 for PsxA (**Supplementary Table 1**). This evidence suggests that the two toxin operons did not diverge since their vertical inheritance from a last common ancestor. Instead, the *psx* locus of *B. pseudohinzii* appears to have been independently acquired horizontally from a currently unknown source.

### *B. pseudohinzii* PsxA Contains Conserved Sites and Predicted Structural Homology to *B. pertussis* PtxA

**Figure 3:**
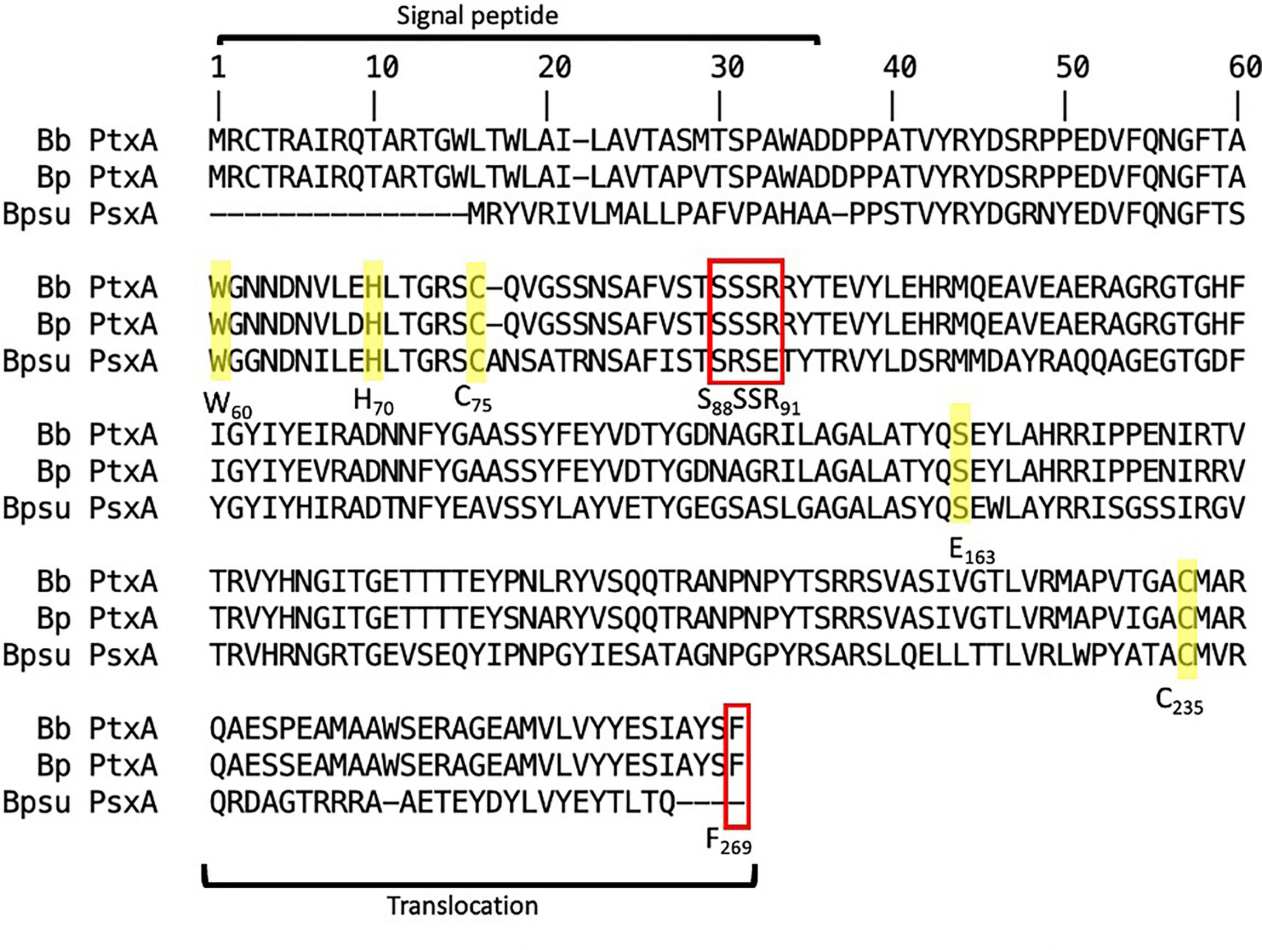
Sequence alignment between Ptx-like toxins of *B*. *pertussis* (Bp), *B*. *bronchiseptica* (*Bb*), and *B*. *pseudohinzii* (*Bpsu*). ADP-ribosyltransferase catalytic sites are notated as H_70_ and E_163._ NAD-glycohydrolase activity sites are notated as C_75_ and W_60_. A-B subunit interaction sites are notated as C_75_ and C_235_. Toxin secretion motif is notated as S_88_SSR_91_. Outer membrane translocation site is notated as F_269_. Red box indicates differences and yellow highlight indicates homology.

To investigate the homology of the *B. pseudohinzii* toxin catalytic unit (PsxA) to the *B. pertussis* toxin catalytic unit (PtxA), sequence alignment and computational modeling were conducted. Despite PsxA only sharing 56% homology to PtxA at the protein sequence level, their ADP-transferase catalytic (H70, E163) (Locht and Antione, 1995) and NAD hydrolase catalytic sites (W60, C75) are conserved (Burns and Manclark, 1989; Cortina and Barbieri, 1989) (**Figure 3**). In addition, the Cys residues involved in disulfate bonding of A-B subunits (C75 and C235) (Burns and Manclark, 1989) are also conserved. Two major differences between PsxA and PtxA reside at the S_88_SSR_91_ secretion motif and the carboxyl-terminal membrane translocation sequence. Compared to PtxA, the Ser89 of PsxA is replaced by an Arg while the Arg91 is substituted with a Glu. It has been experimentally shown that mutation of PtxA Ser89 to Gly led to more than 10-fold decrease in secretion of PtxA (Craig-Mylius et al., 2000). In addition, the carboxyl-terminal membrane translocation motif of PsxA is considerably different from that of PtxA. Notably, the carboxyl-terminal sequence of PtxA, especially the terminal Phe269, was previously shown to be critical for membrane translocation of PtxA (Farizo et al., 2002).

**Figure 4:**
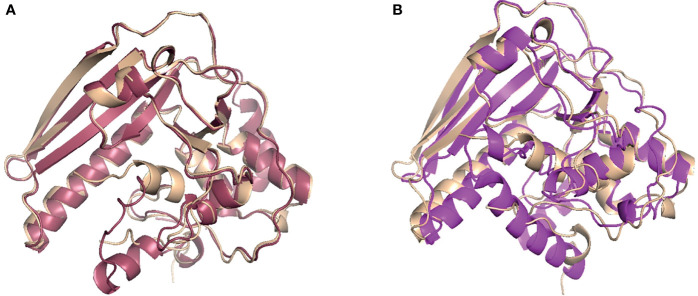
Comparative and *de novo* modeling of PsxA aligned with PtxA. **(A)** Rosetta CM comparative modeling of *B. pseudohinzii* PsxA (pink) based on *B pertussis* PtxA, aligned with *B pertussis* PtxA (tan). RMSD=0.602 Å, confidence= 0.74. **(B)** RoseTTAFold *de novo* modeling of *B. pseudohinzii* PsxA (purple) aligned with *B pertussis* PtxA (tan). RMSD= 1.655 Å, confidence= 0.64.

To computationally model the 3D structure of *B. pseudohinzii* PsxA, two modelling approaches were used, namely *de novo* modelling and comparative modelling. The *de novo* modelling approach utilized is a machine deep learning based method, RoseTTAFold, which is a recent milestone in *de novo* modelling with high accuracy (Baek et al., 2021). The comparative modelling approach used here, RosettaCM, combined four independent modelling methods (Song et al., 2014) and modelled the PsxA structure based on the published crystal structure of *B. pertussis* PtxA (PDB ID: 1prt) (Stein et al., 1994). As shown in **Figure 4**, the predicted structure of PsxA by comparative modelling (pink) or by *de novo* modelling (purple) is aligned with the known structure of PtxA (tan). To evaluate the structural similarities between modelled PsxA and PtxA, RMSD (root-mean-square deviation) was calculated. Predicted structures of PsxA by both methods display high similarities to PtxA with RMSD values of less than 2 Å (RosettaCM RMSD = 0.602 Å, RoseTTAFold RMSD = 1.655 Å). To evaluate the quality of *de novo* modelling, the lDDT score (local Distance Difference Test on All Atoms) was calculated (Hiranuma et al., 2021). Models with IDDT scores higher or equal to 0.6 is considered correct. The RoseTTAFold prediction has an lDDT score of 0.64, indicating qualified prediction.

### PsxA Contributes to Persistence in Middle Ears

**Figure 5:**
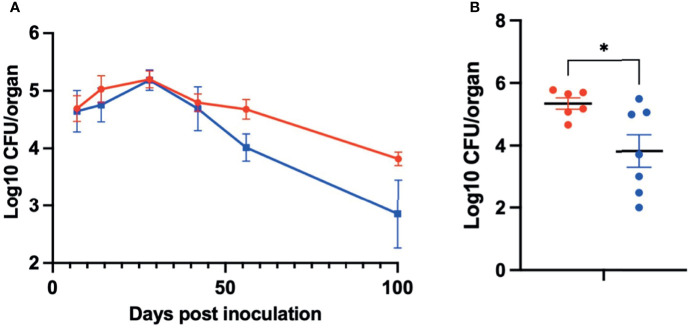
PsxA contributes to persistence of *B. pseudohinzii* in the middle ears. **(A)** Log10 of CFU of WT *B. pseudohinzii* (red) or *B. pseudohinzziΔpsxA* (blue) recovered from the middle ears of C57BL/6 mice on days 7, 14, 28, 42, 56, and 100 post inoculation (n=4 per strain and timepoint). **(B)** Log10 of CFU isolated from the middle ears of C57BL/6 mice on day 56 post inoculation (WT *B. pseudohinzii* n=6 and *B pseudohinziiΔpsxA* n=7). Error bar shows standard error of mean. Statistical significance was calculated *via* Two-way ANOVA. *p < 0.0332.

To determine the role of PSx in the colonization and persistence of *B. pseudohinzii* in the middle ears, an isogenic derivative with an in-frame deletion of the coding region of *psxA* was generated by allelic exchange (**Supplementary Figure 2**). The construct and subsequent integration was verified by Sanger sequencing. Growth curves of the WT and mutant *B. pseudohinzii* were performed simultaneously and no growth defects were observed (**Supplementary Figure 3**). Allelic exchange was performed three times independently to confirm that the effects were not due to random spontaneous mutations (**Supplementary Figure 4**). To assess the impact of PsxA on bacterium-host interactions, C57BL/6 mice were inoculated intranasally with 5 µL PBS containing 150 CFU of either WT *B. pseudohinzii* or *B. pseudohinzii*∆*psxA*. The middle ears were harvested from four mice each at days 7, 14, 28, 42, 56, and 100 post inoculation and bacterial numbers were assessed (**Figure 5A**). Overall, the mutant and WT strains had similar numbers of bacteria at early timepoints, but the average numbers of *B. pseudohinzii∆psxA* were approximately 90% lower than that of the WT strain at 56 and 100 dpi, although inter-mouse variation in multiple comparisons between small numbers (n=4) of animals resulted in differences that did not meet the threshold for statistical significance. A follow-up experiment focusing on 56 dpi with larger numbers of animals confirmed a clear and significant difference, with the *psxA* mutant being present at on average >90% lower numbers than the WT strain (**Figure 5B**). Due to known contributions of biofilm formation in the persistence of bacterial middle ear infections, the biofilm forming abilities of both WT and mutant *B. pseudohinzii* strains were evaluated and no difference was observed between the two strains (**Supplementary Figure 5** **)**. This, along with the prolonged time required to observe a defect reduces the likelihood of PsxA contributing to resistance of innate factors and indicates a role in evading adaptive immune clearance.

### The Adaptive Immune Response Contributes to the Control of Middle Ear Infections

**Figure 6:**
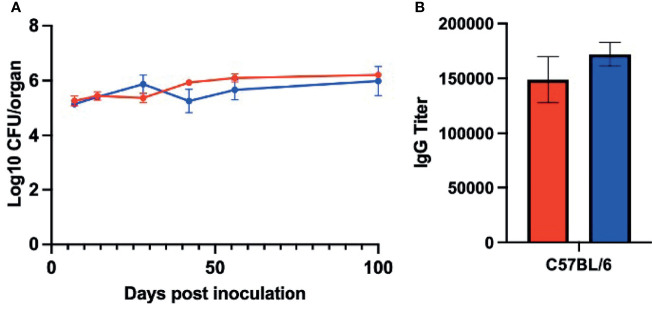
PsxA does not affect persistence in *Rag-/-* mice or serum antibody production. **(A)** Log10 of CFU of WT *B. pseudohinzii* (red) or *B. pseudohinziiΔpsxA* (blue) recovered from the middle ears of Rag1^-/-^ mice on days 7, 14, 28, 42, 56, and 100 post inoculation (n=4 per strain per timepoint). **(B)** Serum from C57BL/6 mice 56 days after inoculation with *B. pseudohinzii* or *B pseudohinziiΔpsxA* was analyzed by ELISA for anti-*B. pseudohinzii* titers (results are the average of two independent assays). Error bars show the standard error of the mean. Statistical significance was calculated *via* Two-way ANOVA.

The observation that there was no defect in the mutant’s ability to colonize, grow, and spread to the middle ears indicate that PSx has no critical roles in these aspects of early infection processes. A defect was only observed after a month of infection, when adaptive immune functions are dominant aspects of the host response, suggesting that PSx may be involved in evading adaptive immune-mediated clearance to allow *B. pseudohinzii* to persist. To determine whether PsxA mediates persistence *via* effects on host adaptive immunity, the persistence of WT *B. pseudohinzii* and *B. pseudohinzii∆psxA* was assessed in mice deficient in mature B and T cells (Rag1^-/-^ mice). Rag1^-/-^ mice were challenged as above and middle ears were harvested at days 7, 14, 28, 42, 56, and 100 days post inoculation and bacterial load was assessed (**Figure 6A**). WT and mutant *B. pseudohinzii* strains similarly infected Rag1^-/-^ mice at all time points, indicating that PsxA is only required for persistence when B and T cells are present and that PSx is not required to resist the anti-bacterial functions of the innate immune cells present in these mice, including macrophages, neutrophils, and NK cells, among others. The defect of the *psxA* mutant was only observed in the presence of adaptive immunity, indicating that PSx facilitates persistence by mediating evasion of some function(s) of mature B and/or T cells.

### PsxA Does Not Affect Production of High Titers of Antibodies

Antibodies are critical aspects of the immune response to *Bordetella spp.* infections and are required for the clearance of both *B. bronchiseptica* and *B. pertussis* from the lower respiratory tract (Kirimanjeswara et al., 2003). The effect of PSx on the antibody response to infection was therefore assessed. Immunocompetent C57BL/6 mice were inoculated with WT or *∆psxA* strains, as above, and serum was collected 56 days post inoculation and serum anti-*B. pseudohinzii* IgG titers were analyzed *via* ELISA. These assays demonstrated that *B. pseudohinzii* and *B. pseudohinzii∆psxA* induced similarly high IgG titers (**Figure 6B**). These observations indicate that PsxA did not substantially disrupt the production of high titer antibodies, and that high titers of antibodies were not sufficient to clear *B. pseudohinzii* infection from the middle ears.

### The psxA Mutant Is More Rapidly Cleared *via* a T Cell Dependent Mechanism

**Figure 7:**
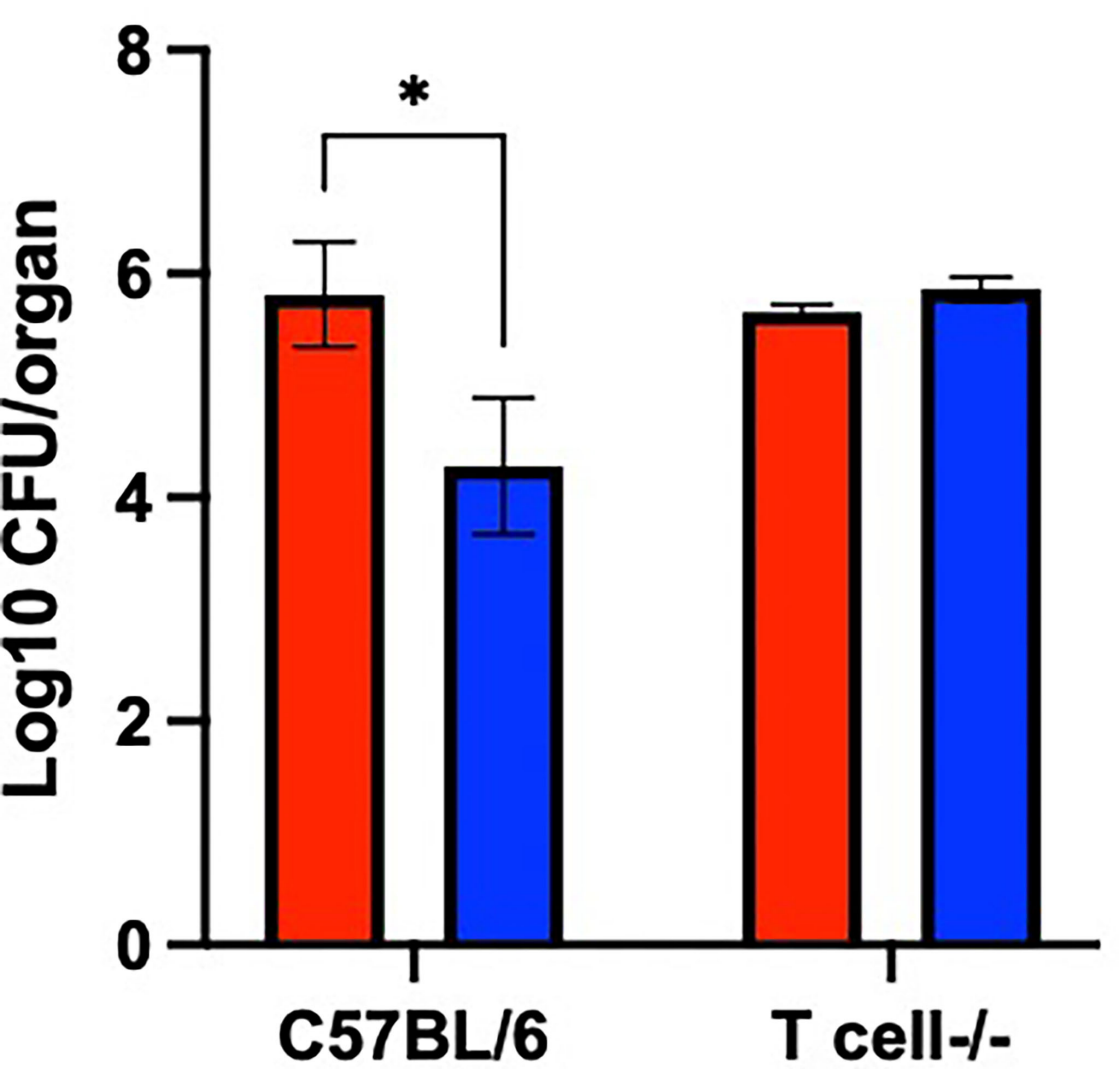
T cell-mediated bacterial clearance is disrupted by PsxA. Log10 of CFU of WT *B. pseudohinzii* (red) or *B. pseudohinziiΔpsxA* (blue) recovered from the middle ears of C57BL/6 or T cell-/- mice at 56 days post inoculation. N=3 per mouse type and bacterial strain, assay was performed in duplicate. Error bars show the standard error of the mean. Statistical significance was calculated *via* Two-way ANOVA. *p < 0.0332.

Based on the results in **Figure 6**, we hypothesized that PsxA contributes to persistence *via* evasion of T cell-mediated immune responses. To test this, the persistence of WT and mutant *B. pseudohinzii* in mice lacking both α/β and δ/γ T cells (T cell^-/-^) was assessed. At 56 dpi, the middle ears of these mice were harvested for bacterial enumeration (**Figure 7**). The WT bacterium was recovered in similar numbers from immunocompetent C57BL/6 and T cell^-/-^ mice, indicating that T cells have a limited role in the control of *B. pseudohinzii* infection of the middle ear. In contrast, the *psxA* mutant was recovered from the middle ears of C57BL/6 mice in significantly lower numbers than the wild type *B. pseudohinzii* (**Figure 5**), but this difference disappeared in mice lacking T cells. Together these results indicate that PsxA mediates persistence by disrupting T cell mediated anti-bacterial effects.

## Discussion

We have limited understanding of the natural processes involved in chronic OM and the bacterial factors that contribute to modulation of the host immune system to allow persistence. Current experimental systems using human pathogens injected in large numbers into the middle ears of mice or chinchillas can model some aspects of severe acute disease and pathology. However, these systems do not encompass critical aspects of natural bacterium-host interactions occurring during initial establishment or persistence of middle ear infections and cannot reveal the mechanisms involved in those. *B. pseudohinzii* has been previously demonstrated to naturally cause middle ear infection in mice that persist for over 3 months, causing chronic otitis media and progressive hearing loss (Dewan et al., 2019). Here, genes particular to *B. pseudohinzii* and identified as a PTx-like factor, PSx, were investigated and modeling of the catalytic component reveals substantial predicted structural homology to PtxA. A *psxA* deletion mutant of *B. pseudohinzii* demonstrated a significant persistence defect, with a 90% reduction in bacteria recovered from the middle ears of immunocompetent C57BL/6 mice at 56 days post inoculation. This defect was ameliorated in mice lacking both B and T cells and mice lacking only T cells, indicating that T cells are required for the dramatic reduction in numbers of the mutant. These results also indicate that PSx contributes to persistence by mediating the evasion of T cell activities that can otherwise reduce bacterial numbers greatly. This experimental system can allow the mechanisms by which T cells reduce bacterial numbers in the middle ear to be further probed.

The amino acid-level sequence homology between PSx and PTx (56%) demonstrates moderate overall conservation between the two AB_5_ toxins. However, the overall homology between the core genomes of *B. pertussis* and *B. pseudohinzii* is much higher, averaging about 80%. The *ptx* loci appears in the very closely related classical bordetellae (*B. pertussis, B. bronchiseptica*, and *B. parapertussis*) but not in any of the more distantly related *Bordetella* species, leading to the conclusion that it was acquired horizontally by this lineage as it emerged as successful mammalian respiratory pathogen (**Supplementary Figure 1**) (Parkhill et al., 2003; Linz et al., 2016). The *B. pseudohinzii* genome appears to have acquired, and inserted at a different genome location, a distantly related version of the entire locus, including homologs of all nine genes (*pltA* through *pltI*) that encode the type IV secretion system (T4SS) required for toxin secretion. The corresponding H values ranged from H=0.241 for PtlE to 0.607 for PltA (**Supplementary Table 1**) and support its having been acquired from some distantly related genus. The presence of this system in the genome of *B. pseudohinzii* indicates this organism contains all the machinery known to be needed for the expression and secretion of an intact holotoxin.

Although overall homology is relatively low, the catalytic sites of PTx and PSx are particularly well conserved both in their sequences and predicted 3-dimensional structure, as they are in most AB_5_ toxins. This indicates likely similarity in their catalytic activities, suggesting PSx acts like PTx, potentially ADP ribosylating G proteins, although the specificity of their substrates might vary. However, there are significant differences between the protein sequences, notably in the secretion motif and carboxyl terminal sequence. These nonhomologous features could be a result of adaptation of differential secretion and membrane translocation machineries by *B. pseudohinzii*. PTx-like toxins encoded by a diverse group of pathogenic bacteria and their functions have been extensively studied. These include species and strains of *E. coli* and *Salmonella*, among others (Littler et al., 2017; Tamamura et al., 2017), and are characterized as an AB_5_ toxin with ADP-ribosyltransferase activity. Importantly, it has been observed that the catalytic A subunit of PTx and PTx-like toxin are conserved though they may not target the same motifs on host G proteins (Littler et al., 2017). Previous observations have identified the multifactorial role of PTx in the activation and subsequent modulation of T cell signaling, leading to T cell death (Schneider et al., 2007). Analysis of the binding motifs of the catalytic A subunit and B subunit could provide insight into the intra- and extracellular host cell motifs that are specifically targeted by PSx.

*B. pseudohinzii* was described as an otopathogen of laboratory mice in 2016 (Ivanov et al., 2016) and has since been isolated from various rodent sources (Loong et al., 2018). Several investigations into this natural colonizer of mice has revealed key functions and characteristics that allow it to colonize and evade immune clearance as an upper respiratory tract pathogen. This includes the impairment of movement and death of ciliated cells (Perniss et al., 2018). This observation provides an explanation for the progressive hearing loss previously observed in *B. pseudohinzii* infected mice (Dewan et al., 2019) and could also contribute to bacterial persistence. Additionally, T cells have been implicated in the control and clearance of both upper respiratory and middle ear infections for various *Bordetella* infections (Leef et al., 2000; Harvill et al., 1999a). Here, mice deficient in all T cell subsets were unable to control or clear both WT and mutant *B. pseudohinzii* infections in the middle ears. The bacterial levels of WT *B. pseudohinzii* recovered from both T cell^-/-^ and immunocompetent mice are similar, indicating these infections were unaffected by the presence or lack of T cells. The *psxA* mutant, however, was able to survive to higher levels in T cell^-/-^ mice compared to immunocompetent C57BL/6 mice. This indicates that this mutant strain was uniquely susceptible to T cell-mediated protection and that PsxA likely contributes to the ability of *B. pseudohinzii* to evade T cell mediated clearance. This resistance to T cell action is consistent with the ability of *B. pseudohinzii* to suppress a Th17-mediated allergic response in an asthma model (Jaeger et al., 2020). In addition, these effects are also consistent with published observations on the interactions between PTx from *B. pertussis* and T lymphocytes as well as other published PTx-like toxins, including inhibition of migration and cytokine signaling (Cyster and Goodnow, 1995; Schneider et al., 2009; Brink et al., 2018). These results support the presentation of PSx as a novel PTx-like toxin that contributes to the persistence of *B. pseudohinzii.*

Since *B. pseudohinzii* is well adapted as an efficient middle ear pathogen of mice, we have the unique opportunity to observe the natural process of colonization of the middle ear and the contributions of virulence factors and toxins in these infections. This is an exceedingly powerful tool for the understanding of both host and pathogen factors that contribute to establishment and persistence of chronic middle ear infections. For example, the T cell mediated mechanisms that reduce the numbers of the *psxA* mutant can be probed to better understand how T cells can contribute to immunity in the middle ear. Further, *B. pseudohinzii* lacking PsxA still persists, albeit at lower levels, whereas *B. bronchiseptica* is completely cleared from the middle ear. This is strong evidence that there are other immune evasion elements in the genome of *B. pseudohinzii*, not shared by *B. bronchiseptica*, that remain to be discovered.

Established models of middle ear infections have contributed substantially to our understanding of factors that contribute to pathogenesis, identifying pneumococcal surface proteins that cause inner ear damage (Schachern et al., 2009) and *in vivo* interactions of *P. aeruginosa* with macrophages and middle ear epithelial cells (Mittal et al., 2014; Mittal et al., 2016). As the incidence and burden of chronic otitis media increases, the field may benefit from continued pursuit of alternative models that can provide new windows through which to observe infection. We present this model of a natural otitis media infection as a complementary experimental system to existing models in order to further our knowledge of the complex bacterium-host interactions involved in chronic otitis media.

## Data Availability Statement

The raw data supporting the conclusions of this article will be made available by the authors, without undue reservation.

## Ethics Statement

The animal study was reviewed and approved by Institutional Animal Care and Use Committees at The University of Georgia at Athens, GA.

## Author Contributions

LM, KD, BL, and EH conceived the study. LM, CS, YS, KD, BL, and EH designed the experiments. LM, CS, YS, and BL performed the experiments. LM, CS, YS, and BL analyzed the data. LM, CS, YS, BL, and EH wrote the manuscript. All authors contributed to the article and approved the submitted version.

## Funding

This work was supported by grants AI149787, DC018496, AI156293, and AI159347 of the National Institutes of Health to EH. The funders had no role in study design, data collection, and interpretation, or the decision to submit the work for publication.

## Conflict of Interest

The authors declare that the research was conducted in the absence of any commercial or financial relationships that could be construed as a potential conflict of interest.

## Publisher’s Note

All claims expressed in this article are solely those of the authors and do not necessarily represent those of their affiliated organizations, or those of the publisher, the editors and the reviewers. Any product that may be evaluated in this article, or claim that may be made by its manufacturer, is not guaranteed or endorsed by the publisher.

## Supplementary Material

Supplementary Figure 1 Structure and chromosomal location of the pertussis toxin-encoding operon in *B. pseudohinzii*. **(A)** Structural comparison of the pertussis toxin operons in *B. pertussis* and *B. pseudohinzii*. The red rectangles and lines show blocks of sequence homology, with a color shading gradient from low (light pink) to high homology (red). The structure of the operon is conserved, with the exception of the duplicated *ptxB/ptxC* genes in the classical *Bordetella* species (*B. pertussis*, *B. parapertussis* and *B. bronchiseptica*). **(B)** Different chromosomal location of the pertussis toxin operon in *B. pseudohinzii* and in the classical bordetellae, here *B. bronchiseptica*.

Supplementary Figure 2 Genetic context of *B. pseudohinzii*∆*psxA* mutant strain.

Supplementary Figure 3 Similar laboratory growth *in vitro* of *B. pseudohinzii* WT (blue) and *B. pseudohinzii*∆*psxA* (orange) bacteria. There were 3 technical replicates in each time point per group. Error bars show the standard error of mean. Statistical significance was calculated using Unpaired t-test. *p < 0.05, **p < 0.01, ***p < 0.001, n.s. > 0.05.

Supplementary Figure 4 Similar bacterial recovery of *B. pseudohinzii∆psxA* isogenic mutants from ears. Bacterial recovery of mutant replicates (I-23, I-13, and I-10) from the middle ears 56 dpi from C57BL/6 mice. The mutant here referred to as I-23 was utilized for all experiments with *B. pseudohinzii∆psxA*. Statistical significance was calculated using One-way ANOVA. *p<0.05, **p<0.01, ***p<0.001, n.s. >0.05.

Supplementary Figure 5 Biofilm forming abilities of WT and mutant *B. pseudohinzii.* WT *B. pseudohinzii* (WT Bpsu) (red) and *B. pseudohinzii∆psxA* (Bpsu∆psxA) (blue) demonstrate similar biofilm forming capabilities after 48 hours in PBS. There are three replicates per group. Statistical significance was calculated using Unpaired t-test. *p < 0.05, **p < 0.01, ***p < 0.001, n.s. >0.05.

Supplementary Table 1
*B. pseudohinzii* and *B. pertussis* protein homology. Comparison of amino acid sequences of PSx (*B. pseudohinzii*) and PTx (*B. pertussis*) toxin subunits and Type IV secretion system subunits.
