# Supplementary figures and images for: Contribution of a Novel Pertussis Toxin-Like Factor in Mediating Persistent Otitis Media
Source: Front Cell Infect Microbiol. 2022 Mar 11;12:795230. doi: 10.3389/fcimb.2022.795230 (PMC8963424; doi:10.3389/fcimb.2022.795230)

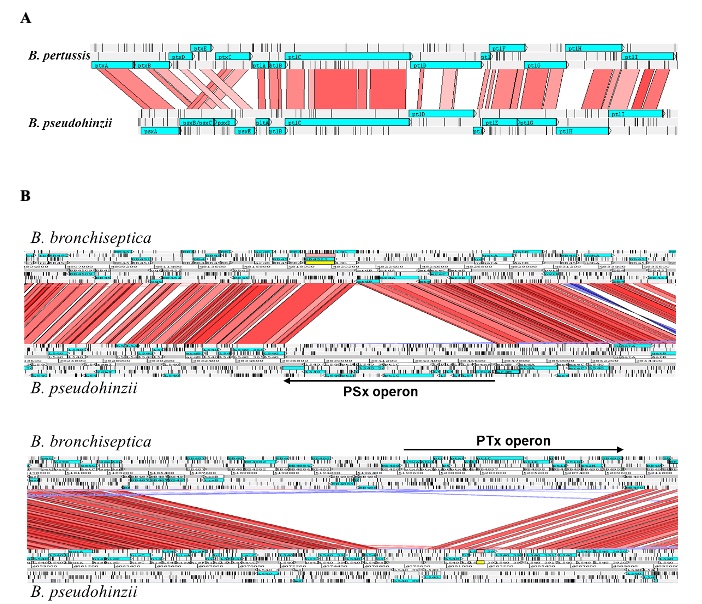

Supplement: Supplementary Figure 1 — Structure and chromosomal location of the pertussis toxin-encoding operon in B. pseudohinzii. (A) Structural comparison of the pertussis toxin operons in B. pertussis and B. pseudohinzii. The red rectangles and lines show blocks of sequence homology, with a color shading gradient from low (light pink) to high homology (red). The structure of the operon is conserved, with the exception of the duplicated ptxB/ptxC genes in the classical Bordetella species (B. pertussis, B. parapertussis and B. bronchiseptica). (B) Different chromosomal location of the pertussis toxin operon in B. pseudohinzii and in the classical bordetellae, here B. bronchiseptica. [file Image_1.jpeg]

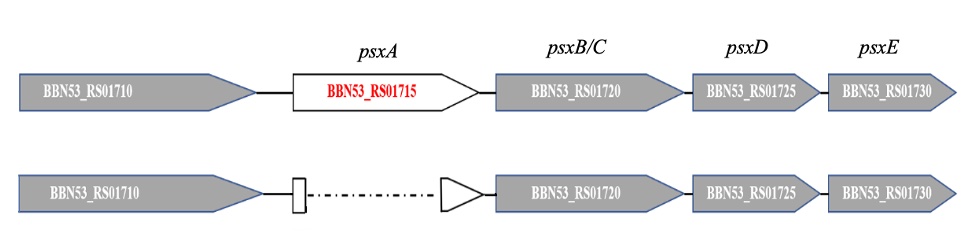

Supplement: Supplementary Figure 2 — Genetic context of B. pseudohinzii∆psxA mutant strain. [file Image_2.jpeg]

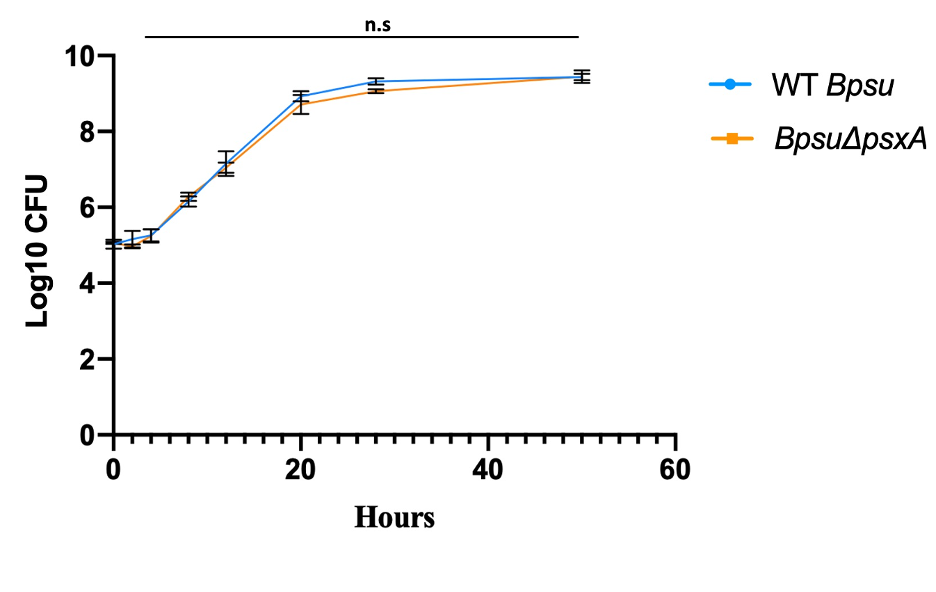

Supplement: Supplementary Figure 3 — Similar laboratory growth in vitro of B. pseudohinzii WT (blue) and B. pseudohinzii∆psxA (orange) bacteria. There were 3 technical replicates in each time point per group. Error bars show the standard error of mean. Statistical significance was calculated using Unpaired t-test. *p < 0.05, **p < 0.01, ***p < 0.001, n.s. > 0.05. [file Image_3.png]

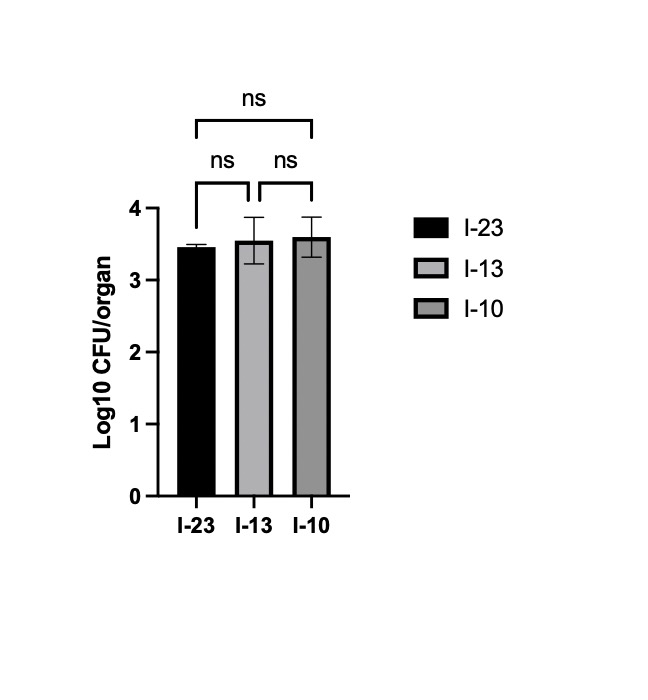

Supplement: Supplementary Figure 4 — Similar bacterial recovery of B. pseudohinzii∆psxA isogenic mutants from ears. Bacterial recovery of mutant replicates (I-23, I-13, and I-10) from the middle ears 56 dpi from C57BL/6 mice. The mutant here referred to as I-23 was utilized for all experiments with B. pseudohinzii∆psxA. Statistical significance was calculated using One-way ANOVA. *p<0.05, **p<0.01, ***p<0.001, n.s. >0.05. [file Image_4.jpeg]

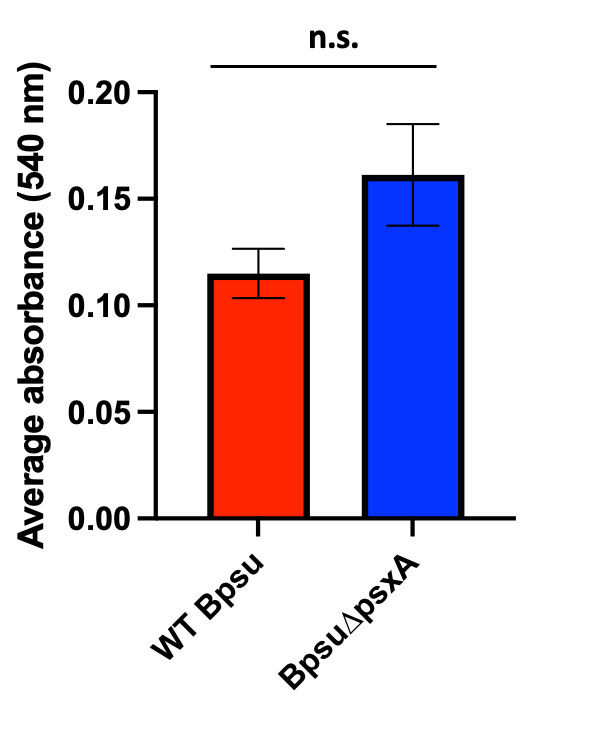

Supplement: Supplementary Figure 5 — Biofilm forming abilities of WT and mutant B. pseudohinzii. WT B. pseudohinzii (WT Bpsu) (red) and B. pseudohinzii∆psxA (Bpsu∆psxA) (blue) demonstrate similar biofilm forming capabilities after 48 hours in PBS. There are three replicates per group. Statistical significance was calculated using Unpaired t-test. *p < 0.05, **p < 0.01, ***p < 0.001, n.s. >0.05. [file Image_5.png]

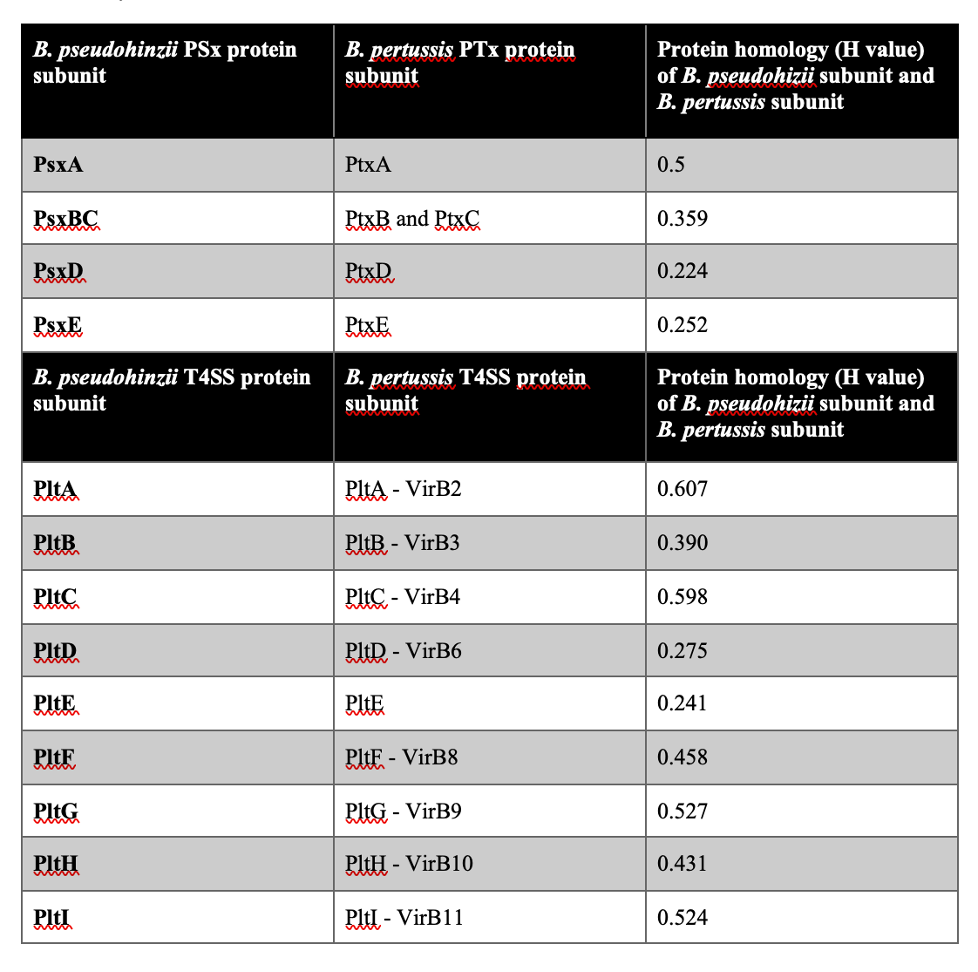

Supplement: Supplementary Table 1 — B. pseudohinzii and B. pertussis protein homology. Comparison of amino acid sequences of PSx (B. pseudohinzii) and PTx (B. pertussis) toxin subunits and Type IV secretion system subunits. [file Image_6.png]
